# Fungal endophytes of cactus (*Stenocereus* spp.) as a potential alternative to alleviate drought stress in juveniles of *Theobroma cacao* L. ICS95

**DOI:** 10.1128/msphere.00865-25

**Published:** 2026-04-13

**Authors:** Karen Sofía Trujillo-Ortigoza, Angelis Marbello-Santrich, Juliana González-Tobón, Fermín Rada, Marcela Guevara-Suarez, Silvia Restrepo

**Affiliations:** 1 Laboratorio de Micología y Fitopatología, Universidad de los Andes 27991, Bogotá, Colombia; 2 Applied Genomics Research Group, Vicerrectoría de Investigación y Creación, Universidad de los Andes, Bogotá, Colombia; 3 Boyce Thompson Institute, Ithaca, New York, USA; 4 Laboratorio de Ecología y Fisiología Vegetal (EcoFiV), Departamento de Ciencias Biológicas, Universidad de los Andes, Bogotá, Colombia; 5 Instituto de Ciencias Ambientales y Ecológicas (ICAE), Universidad de Los Andes, Mérida, Venezuela; Max-Planck-Institut fur Biologie Tubingen, Tuebingen, Germany

**Keywords:** drought, endophytes, leaf water potential, proline, water stress, xerophytic

## Abstract

*Theobroma cacao*, one of Colombia’s most socially significant crops, faces productivity challenges due to drought. This stress can reduce growth, leaf area, and stomatal conductance (*K*_*s*_) and generate reactive oxygen species. Therefore, exploring solutions to enhance drought tolerance is crucial. This study aimed to (i) design a strategy to select fungal root endophytes with the potential to alleviate drought stress in plants; (ii) isolate fungi from *Stenocereus* spp. to induce drought tolerance in *T. cacao* genotype ICS95. *In vitro* drought tolerance screening identified five fungal isolates that exhibited the highest biomass production and less than 20% biomass loss under drought conditions compared with non-drought conditions. The soil of juvenile *T. cacao* plants was inoculated with these isolates, and physiological and morphological parameters were assessed, including leaf water potential (Ψ_L_), *K*_*s*_, proline content, and growth. The results showed a significant decrease in Ψ_L_ and *K*_*s*_ in juveniles under drought stress, which was observed across all five fungal isolates tested. However, juveniles inoculated with *Phoma* sp. exhibited significantly less negative Ψ_L_ than non-inoculated controls, suggesting that this fungus may be a potential inducer of drought tolerance in *T. cacao* ICS95. One intriguing result was that plants inoculated with this fungus accumulated less proline during the drought treatment. Under non-drought conditions, juveniles inoculated with *Fusarium* sp. exhibited a significant increase in specific leaf area under non-drought conditions compared to non-inoculated control. These findings suggest that fungal endophytes associated with *Stenocereus* spp. affect the physiological response of cacao juveniles under drought conditions.

**IMPORTANCE:**

*Theobroma cacao* is among the world’s most valuable crops, yet its productivity is increasingly threatened by fluctuating rainfall and prolonged drought. Identifying sustainable strategies to mitigate these impacts is therefore critical. Xerophilic plants, such as *Stenocereus* spp., harbor diverse fungal endophytes adapted to arid environments, representing a promising source of microorganisms capable of enhancing stress tolerance in commercial crops. Our study demonstrates that cactus-derived endophytes could improve drought resilience in juveniles of cacao, in particular, the fungal endophyte *Phoma* sp. maintained less negative leaf water potential values under drought stress conditions and exhibited significantly lower proline accumulation compared to non-inoculated controls. Furthermore, under favorable conditions, some endophytes could promote growth by increasing leaf area compared to non-inoculated plants. These findings underscore the potential of fungal endophytes from arid ecosystems as biotechnological tools for sustainable cacao production. Further studies should explore the role of fungi when affecting plant proline metabolism.

## INTRODUCTION

*Theobroma cacao* L., commonly known as cacao, is a native crop of the Amazon basin. This crop plays a significant commercial role in Africa, Central America, and South America (1, 2). Cocoa beans are used to produce chocolate, pharmaceutical formulations, cosmetic products, and alcoholic beverages (1–3), making the crop a valuable commodity across various industries.

Colombia is one of the largest producers of *T. cacao*, renowned for producing fine, aromatic, and high-quality grains (4, 5). This country’s main cocoa-producing regions include Santander, Antioquia, Arauca, Tolima, and Huila. In 2020, its production generated 165,000 direct and indirect jobs in Colombia, making it one of the primary sources of income for over 52,000 families (4). Furthermore, cocoa has been established as an alternative to peace efforts in post-conflict territories and as a substitute for illicit crops (4). This crop is therefore considered to play an essential role in Colombia’s social and economic development.

However, climate change can affect cocoa production, primarily because of its sensitivity to abiotic stresses such as drought and salinity (1). This crop requires temperatures between 18°C and 32°C, 1,500 and 2,500 mm of rainfall, and constant humidity for optimal growth (5, 6). A 2024 report by the United Nations indicates an increase in the frequency of daily heatwaves and drought months in recent decades, which may lead to a decline in agricultural production and heightened food insecurity (7). Additionally, a 2024 report from the Colombian Institute of Hydrology, Meteorology, and Environmental Studies predicts variable drought events across the country’s cocoa-producing regions (8). For departments such as Antioquia and Huila, an increase in precipitation is expected; however, in Arauca, Santander, and specific areas of Tolima, a decrease in rainfall is anticipated, resulting in longer drought seasons (8). Considering that cacao seedlings and juveniles may be more susceptible to drought due to their shallow rooting depth, which ranges from 0.4 to 0.8 m, water absorption may be severely limited during extended drought periods (1, 9). This, in turn, adversely affects nutrient uptake, water relations, and gas exchange. As a result, these conditions may lead to alterations in the crop’s growth, biomass, and various biochemical characteristics, ultimately impacting cocoa productivity and quality (9).

Different strategies have been proposed given the effects of drought on cocoa production. One potential strategy is using endophytic fungi (10), which are microorganisms that live inside the plant without causing apparent damage (11) and, instead, can confer several benefits to the plant, including protection against herbivory and plant pathogens, improved growth, protection, and tolerance to abiotic stress (12, 13). Endophytes can stimulate plant responses, such as producing reactive oxygen species (ROS) enzymes and increasing phytohormone levels, which promote root and shoot growth (14, 15). For example, Bae et al. (16) demonstrated that the fungal endophyte *Trichoderma hamatum* isolated from *Theobroma gileri* promotes growth and delays drought stress responses in *Theobroma cacao*. Consequently, fungal endophytes could represent an important alternative to enhance the overall fitness of the plant under drought conditions (10). However, it is essential to note that only 1% of environmental microorganisms have been identified, underscoring the need for a deeper understanding of the diversity of microorganisms, such as endophytes (17). These microorganisms represent a group with great potential, and further exploration could uncover new possibilities for their application in agriculture. Studies on microbial diversity in soil and endophytes have shown their importance. For instance, research by Erktan et al. (18) suggests that a high diversity of these microorganisms increases the likelihood of discovering species with various potential agricultural applications, such as nutrient assimilation, biocontrol, growth promotion, protection against abiotic stress, and water content regulation, among others.

Xerophytic angiosperms, exemplified by cacti, have been identified as hosts to endophytes. These endophytes have been suggested to mitigate abiotic stresses, such as salinity and drought, and fostering the growth of host plants (19). According to studies on drought tolerance in tomato and cucumber, such as the one developed by Miranda et al. (20), tomato plants inoculated with endophytes from *Eragrostis cilianensis* showed optimal tolerance to drought stress. These plants exhibited lower levels of ROS enzymes, higher stomatal conductance (*K*_*s*_), and increased root biomass, among other beneficial effects, compared to the control group.

The broad host range of fungal endophytes isolated from cacti positions them as promising candidates for agricultural applications (21). According to previous studies, these microorganisms demonstrate a considerable host range, capable of colonizing diverse plant species across different genera and families without exhibiting strict host specificity (22, 23). Furthermore, endophytes isolated from *Ixeris repenes*, a coastal plant native to East Asia, produce bioactive secondary metabolites, including active gibberellins, which confer drought tolerance in rice (24). These findings suggest a direct and indirect role through which endophytes from stress-adapted plants can enhance stress resilience in commercial crops.

However, the potential application of cacti-derived endophytes remains largely unexplored, particularly for xerophytic genera such as *Stenocereus* spp., despite their wide distribution throughout Colombia (25). This study aimed (i) to design a novel strategy to select fungal endophytes with the potential to alleviate drought stress in plants; (ii) to evaluate the potential of root endophytes from *Stenocereus* spp. to alleviate drought stress in *Theobroma cacao* ICS95 juveniles through soil-based inoculation assays.

## MATERIALS AND METHODS

### Endophytic fungal isolation and *in vitro* drought stress selection

#### Study site and sampling

Endophytes were sampled in July 2023 in two localities in Colombia: The Tatacoa Desert of the municipality of Villavieja, Huila department, and Taganga, Magdalena department (see all geographical coordinates in Table S1). At each location, roots were collected from 12 mature cactus *Stenocereus* spp. specimens at depths ranging from 1 to 20 cm, with a minimum separation distance of 7 m between sampling points. Following the ensuing collection, specimens were hermetically sealed and stored at 10°C for 72 h (26). Subsequent processing for endophytic fungi isolation occurred at the Laboratory of Mycology and Phytopathology of the Universidad de los Andes (Bogota, Colombia). Soil samples (two samples per locality) were collected at a depth of 5 cm from each locality, Tatacoa and Taganga. Laboratory analyses were performed to determine salinity and pH (19).

#### Isolation of fungal endophytes

A systematic procedure was implemented to isolate endophytic fungi from the roots of each plant, involving a thorough wash with tap water to remove any residues of plant material and soil. Subsequently, a disinfection process was done following the protocol by Silva-Hughes et al. (26). This involved immersing each sample in 70% ethanol for 1 min, followed by a 3 min wash with 2% hypochlorite and a subsequent rinse with sterile distilled water (SDW). Noteworthy modifications to the protocol included an additional wash with 70% ethanol after hypochlorite treatment, followed by a final rinse with SDW. The roots were meticulously dried using sterile absorbent paper after the washing procedure.

To assess the efficacy of the procedure, disinfected roots were imprinted on petri plates with potato dextrose agar supplemented with chloramphenicol (500 mg/L; PDA-C) (27). Subsequently, 0.5 cm segments were excised from each sample of disinfected roots, and five fragments were placed on petri plates containing PDA-C. The plates were incubated for up to 7 days at 30°C. This entire process was replicated in triplicate for each plant (26). An axenic culture of each fungus from the root was then established in PDA-C and further incubated at 25°C (26). Isolation rate (IR%) of fungal endophytes from *Stenocereus* sp. was calculated with a modification of the methodology described by Khadka, using equation 1 (28), where Ni represents the number of segments from which fungal isolates were successfully isolated and Nt represents the total number of plant segments incubated.


(1) $$IR\%=\frac{Ni}{Nt}\times 100.$$

#### Identification and diversity of fungal endophytes

The fungal endophyte isolates were grouped into morphotypes, or genera, when possible, based on their morphological description and molecular identification. For the morphological description, the colony of each isolate was described based on its macroscopic characteristics, including surface texture, color (color code: https://color.bio/color-picker), aerial mycelium, and structure production.

Detailed microscopic descriptions were performed when possible, characterizing only those exhibiting reproductive structures, following the dichotomous keys of Cepero et al. (29), Carmichael (30), and Seifert and Gams (31), while isolates with only sterile mycelia could not be fully described morphologically. All morphotypes were molecularly identified, and all isolates were preserved in vials with sterile water.

For molecular identification, fungal isolates were grown on PDA for 5–8 days at 25°C. DNA was extracted using the modified SDS method of Nataraja et al. (32): the lysis was performed in an Eppendorf tube with beads and 500 µL of lysis buffer (100 mM Tris, pH 8.0, 50 mM EDTA, and 1% SDS). One gram of the colony was added and then subjected to a bead beater (Mini-Beadbeater-16) for 40 s. For purification, 275 µL of ammonium acetate (7 M, pH 7.0) and 500 µL of chloroform were added, with an incubation of 40 min at 68°C between the two components. Last, DNA precipitation was achieved by adding 1 mL of isopropanol and incubating for 24 h, followed by a final wash with 1 mL of 70% ethanol. The extracted DNA was eluted with 50 µL of Milli-Q water and stored at −20°C.

All isolates were identified using the internal transcribed spacer region of the rRNA (ITS) with the universal primers ITS1 and ITS4 (33). For the *Fusarium* genus, we also used the translation elongation factor 1-alpha (*Tef-1a*) (34). Single-band PCR products were sequenced using an ABI 3500XL DNA analyzer in the Sequencing Core Facility (Gencore) at the Universidad de Los Andes (Bogotá, Colombia). Sequence assembly and editing were performed using Geneious software v. 2022.2.2 (https://www.geneious.com). Preliminary identification of the isolates to the genus level was performed by analyzing gene sequences, using the BLASTn algorithm with default parameters and choosing the type organisms (35).

#### Selection of fungi based on their *in vitro* tolerance to drought

For the *in vitro* drought tolerance assay, 22 purified morphotypes were selected based on PDA and propagule production growth rates. The strains were incubated on PDA medium at 25°C. Two plugs of the actively growing endophytic fungi were used to inoculate potato dextrose broth (PDB) supplemented with polyethylene glycol (PEG-6000) at a concentration of 20% wt/mL, which generates an osmotic pressure of −0.6 MPa (36). Additionally, as a control, two plugs of the actively growing endophytic fungi were used to inoculate a PDB medium without PEG-6000. Subsequently, all the cultures were incubated in a shaker for 7 days at 28°C and 160 rpm (36). The experiment was performed in triplicate for each morphotype.

After incubation, fungal biomass was measured according to a modified protocol (36). First, the cultures were vacuum filtered through pre-weighed Whatman filter paper (Grade 5). Subsequently, the collected biomass was dried at 70°C for 48 h in a forced-air oven (Memmert UM300) until a constant weight was achieved. The dry biomass of both the control (PDB without PEG-6000) and drought-stressed (PDB with PEG-6000) cultures was then quantified by weighing it. Finally, the percentage of biomass loss (equation 2) was calculated for each morphotype to determine drought tolerance.

Fungal strains exhibiting less than 20% biomass loss under drought conditions were selected for *in vivo* assays. Kruskal–Wallis tests followed by Dunn’s *post hoc* test with Bonferroni correction were used to compare differences between the biomass of the morphotypes. All analyses were conducted in R software version 4.4.3 (37) with *P* < 0.05 as the significance threshold.


(2) $$\%Biomass\;loss=\frac{\left(mean\;biomass\;PDB-mean\;biomass\;PEG\;6000\right)}{mean\;biomass\;PDB}\times 100.$$

### *In vivo* evaluation of drought stress

Based on the previous *in vitro* drought tolerance evaluation, five fungi were selected due to their higher biomass production in PDB-PEG6000 and less than 20% biomass loss. The five chosen fungi were used to evaluate drought tolerance in 5-month-old juvenile *T. cacao* plants under greenhouse conditions at Pitalito-Huila (1°52'07.3"N 76°03'50.8" W) during June to July of 2024. Abiotic conditions: air temperature, soil water content (SWC), relative humidity (RH), and photosynthetically active radiation (PAR) were monitored in the greenhouse using the corresponding sensors attached to a HOBO datalogger (model H21, Onset). The experimental design used for this study was randomized. The 5-month-old cocoa juvenile plants, with an average height of 37.4 cm and approximately 12 leaves, were obtained from Maloka Cacao Nursery, located in Rivera-Huila. They were planted in black polyethylene bags (15 × 30 cm and 63 mm caliber) with 2 kg of non-sterile substrate (6 soil:1 husk:1 ash). This standardization of initial conditions, uniform plant age, height, leaf number, and identical soil composition across all experimental units allowed for the direct attribution of any measured effects to the fungal endophyte treatment.

A total of 180 juvenile plants were used, with 90 assigned to the drought treatment (15 per five fungal inoculation treatments, and 15 for a control with SDW supplemented with 1% Tween 80) and 90 to the non-drought control. A culture grown on PDA and oatmeal medium at 25°C for 15 days of each morphotype was used to prepare the inoculum suspension. The spore suspension was adjusted to 1 × 10^6^ conidia/mL in SDW supplemented with 1% Tween 80 (38, 39). The substrate was irrigated with 125 mL of fungal inoculum per 2 kg substrate for each inoculation treatment.

Over 6 days, plants were acclimated in the greenhouse and irrigated with tap water at 7:00 a.m. On day 6, the plants were inoculated with the different fungal strains, and 3 days post-inoculation (day 9), the drought stress assay commenced. A total of 15 plants per fungal morphotype and non-inoculated plants were exposed to drought stress for 14 days (days 10–23), during which they began to exhibit apparent symptoms and signs of drought-related damage. After 14 days (day 23), the plants were re-irrigated with tap water under normal conditions for 2 weeks. The plants in the non-drought control group continued to be irrigated with tap water at 07:00 h, three times a week.

#### Physiological parameters

Leaf water potential (Ψ_L_) and *K*_*s*_ were measured on mature leaves located below the third node of juvenile plant samples, between 11:00 and 14:00 h, at three time points during the experiment: (i) three plants from each treatment, before the drought cycle began (6 days before inoculation); (ii) five plants from each treatment, 14 days after the drought stress cycle started, and (iii) three plants from each treatment, 2 weeks after the plants were re-irrigated. Ψ_L_ was measured following the protocol outlined by dos Santos et al. (40) using a pressure chamber (model 1505D-EXP, PMS Instruments). *K*_*s*_ was measured with a leaf porometer (SC-1, Decagon Devices) (41). Kruskal–Wallis tests followed by Dunn’s *post hoc* test were used to compare physiological parameters among treatments within each condition. To assess differences between conditions (drought vs. non-drought control), Mann–Whitney *U* tests were performed for each treatment separately. All analyses were conducted in R software version 4.4.3 (37) with *P* < 0.05 as the significance threshold.

#### Morphology and growth

Plant height, number of leaves, specific leaf area (SLA), leaf area, and biomass were measured at three time points as described previously. Plant height, number of leaves, and leaf area were measured for each treatment according to the protocols described in references 39, 42. Biomass was determined according to the modified protocol of dos Santos et al. (40) in which the roots, stems, and leaves of the plants of each treatment were stored, separated in paper bags, and oven dried at 75°C (Memmert UM300) for 72 h until constant dry weight (g). Furthermore, the relative growth rate (RGR) was determined according to equation 3 (43), where *S*_1_ and *S*_2_ correspond to initial and final biomasses, respectively, at times 1 (*t*_1_) and 2 (*t*_2_).


(3) $$RGR=\left(\frac{\left(In\;\left(S_{2}\right)\right)-\left(In\;\left(S_{1}\right)\right)}{t_{2}-t_{1}}\right).$$

On the other hand, according to Roderick et al. (44), SLA (cm^2^/g) was determined by collecting the third fully developed leaf with a similar leaf index color. Subsequently, the leaf samples were wrapped in aluminum foil and stored until processed (45). ImageJ software measured leaf area (cm^²^) (46). Leaves were then oven-dried at 70°C (Memmert UM300) for 72 h until constant dry weight (45).

#### Proline

The activity of free proline (µg/mg) was quantified from one fully expanded leaf per plant, with three biological replicates per treatment under both drought and non-drought conditions at the beginning of the experiment (day 0) and after 14 days of exposure to both drought and non-drought control conditions (day 23). A calibration curve was performed using known proline content to ensure accurate quantification. The proline content was determined from healthy leaves of *T. cacao* juvenile plants following the methods described by Bates et al. (47) and dos Santos et al. (40). Healthy leaves of *T. cacao* ICS95 were stored at –80°C until extraction. The leaf tissue was lyophilized for 48 h, after which it was ground in liquid nitrogen. Approximately 35 mg of plant material was mixed with 1 mL of 3% sulfosalicylic acid, followed by agitation in a shaker at 240 rpm for 30 min and centrifugation at 14,000 × *g* for seven min. Subsequently, 0.2 mL of the crude extract was transferred to an Eppendorf tube along with 0.2 mL of acid ninhydrin (1.25 g ninhydrin, 30 mL glacial acetic acid, and 20 mL 6 M phosphoric acid) and 0.2 mL of glacial acetic acid (40, 47). The mixture was vortexed for 15 s. The Eppendorf tubes were incubated in a water bath at 85°C for 120 min. Immediately afterward, the reaction was stopped by placing the tubes in an ice bath for 20 min. Finally, 0.4 mL of toluene was added to the tubes and vortexed for 15 s (40). The samples were left at room temperature to allow phase separation. Absorbance readings were taken at 520 nm using a spectrophotometer (Elisa SpectraMax M4), in triplicate, with toluene as the blank (40). Data normality was assessed using Shapiro-Wilk tests. Mann-Whitney *U* tests compared drought versus non-drought conditions. Within-condition treatment comparisons used analysis of variance (ANOVA) with Tukey’s *post hoc* test for drought conditions, and Kruskal-Wallis with Dunn’s *post hoc* test for non-drought conditions. All analyses were conducted in R software version 4.4.3 (37) with *P* < 0.05 as the significance threshold.

## RESULTS

### Five endophytic fungi from cacti showed remarkable tolerance to drought *in vitro*

#### Soil conductivity and pH

Soil samples collected from the Tatacoa locality (*n* = 2) showed electrical conductivity (EC) values of 30 and 69 ppm, with pH values of 9.6–9.9. In contrast, samples of Taganga locality (*n* = 2) exhibited EC values of 118 and 119 ppm and pH values of 8.8 and 8.9.

#### Fungal morphotypes

A total of 308 fungal endophytes (*n* = 170 for Tatacoa and *n* = 138 for Taganga) were isolated. The IR% for Tatacoa was 94% and Taganga was 76.66% indicating high endophyte colonization at both localities. Furthermore, 49 morphotypes were identified in both localities; 12 were exclusive to Tatacoa and six to Taganga. All morphotypes were identified based on their phenotypic characters (Table S2). Molecular identification also revealed the presence of 49 potential different taxa (Table S2). Of these, 31 were found in both localities, while 12 were exclusive to Tatacoa and six to Taganga. Among the genera identified based on morphological and molecular characteristics in both localities, prominent taxa included *Alternaria* sp., *Aspergillus* spp., *Curvularia* spp., *Ectophoma* sp., *Epicoccum* sp., *Fusarium* spp., *Lasiodiplodia* sp., *Macrophomina* sp., *Moniliophthora* sp., *Monosporascus* sp., *Phoma* sp., *Rhizopus* sp., and *Trichoderma* spp. Furthermore, the genera *Acrophialophora* sp., *Latorua* sp., *Myrmaecium* sp., *Polyporus* sp., and *Rhodotorula* sp. were uniquely found in the Taganga locality. In contrast, *Acrocalymma* sp., *Clavulina* sp., *Coprinellus* sp., *Deniquelata* sp., *Didymella spp*., *Diaporthe* spp., *Didymocrea* sp., *Oblongocollomyces* sp., *Paraboeremia* sp., *Phoma* sp., and *Purpureocillium* sp. were exclusively identified in the Tatacoa locality.

#### Cacti endophytes tolerant to drought

**Fig 1:**
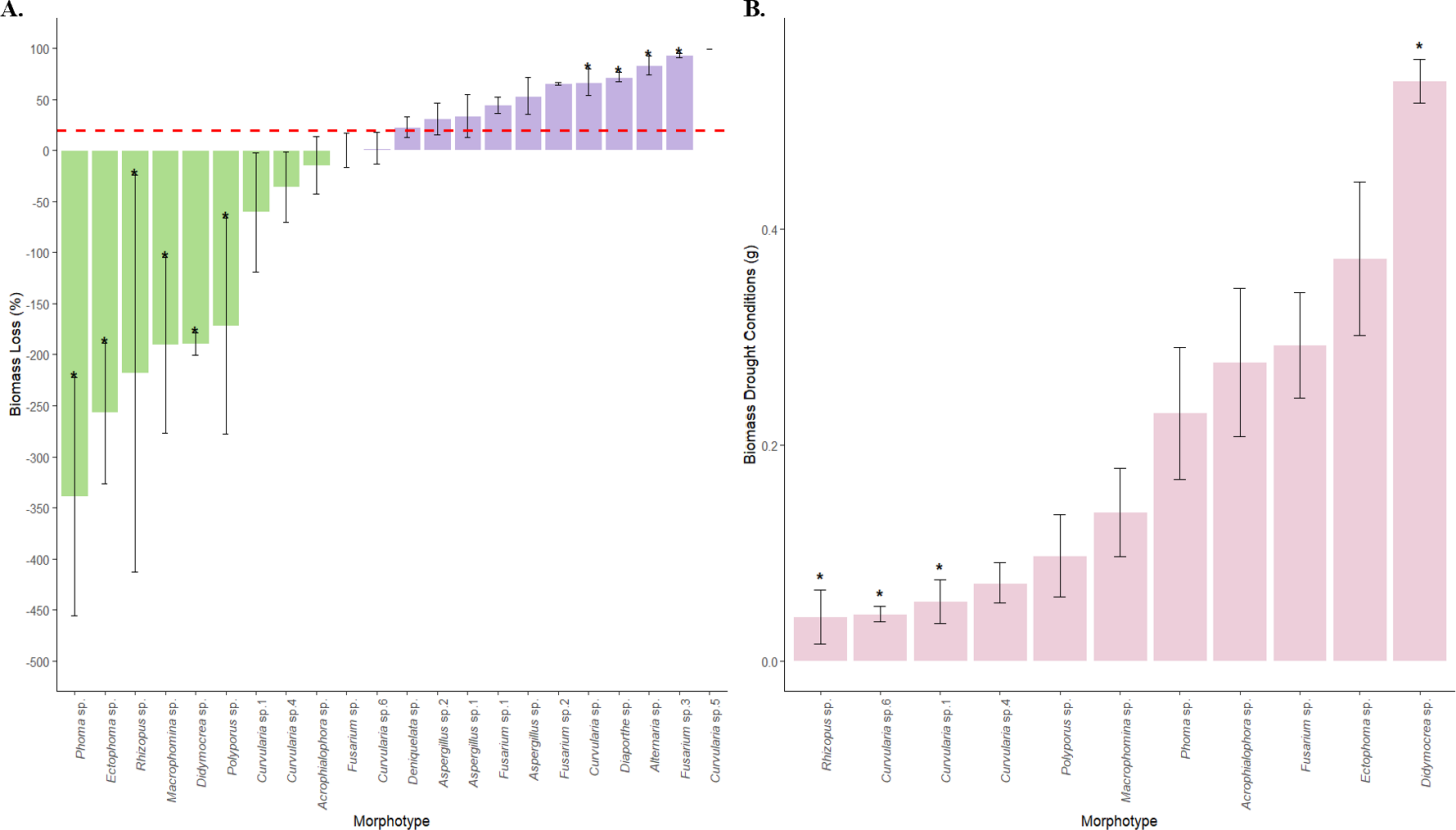
Biomass analysis of fungal endophytes under normal conditions (PDB) and drought conditions (PDB + PEG 6000). (**A**) Percentage of biomass loss of fungal endophytes. The horizontal red line shows a reduction in biomass of 20%. (**B**) Biomass of the 11 morphotypes that showed less than 20% biomass loss under drought conditions. Bars show mean ± SE. Asterisks denote statistical significance among morphotypes based on Kruskal–Wallis followed by Dunn’s test (*P* < 0.05).

Twenty-two taxa were selected for an *in vitro* drought assay based on growth rates and the production of propagules (Fig. 1). Of the 22 fungal strains tested, 11 experienced a biomass loss of less than 20% (red line in Fig. 1A). Nine of these 11 strains showed higher biomass under drought conditions compared to normal conditions. In particular, seven morphotypes (*Curvularia* sp., *Ectophoma* sp., *Didymocrea* sp., *Macrophomina* sp., *Phoma* sp., *Polyporus* sp., and *Rhizopus* sp.) showed significantly lower biomass loss in comparison to the rest of the morphotypes (*P*-value < 0.05; α = 0.05). Subsequently, the biomass of the 11 morphotypes under drought conditions was evaluated (Fig. 1B). Based on these results, five strains (*Acrophialophora* sp., *Didymocrea* sp., *Ectophoma* sp., *Fusarium* sp.1, and *Phoma* sp.) were selected for the greenhouse drought assay because they showed the highest biomass under drought conditions and experienced less than 20% biomass loss (Fig. 1).

### Cactus endophytic fungi promoted plant growth and alleviated drought stress in *T. cacao* ICS95

#### Soil water content

Before the drought stress treatment, plants started with similar volumetric water (0.1121–0.1561 m³/m³) (day 0). After 10 days of drought (day 19), the SWC of juvenile plants exposed to drought remained constant for the last 4 days (less than 0.05 m³/m³) of the drought stress assay (Fig. S1). After re-watering for 11 days, the SWC of juvenile plants under drought conditions increased, reaching values of 0.1252–0.1275 m³/m³ (Fig. S1).

#### Greenhouse conditions

The average temperature inside the greenhouse was 26.5°C, the RH was maintained at 72.90%, and the PAR level averaged 147.042 µmol/m²/s (Table S3). However, temperature and RH varied significantly during the periods when Ψ_L_ and *K*_*s*_ were assessed (0, 23, and 34 days). Temperature and RH were higher after 11–14 days of drought exposure (days 21, 22, and 23) and following the 2-week rehydration period (day 34) compared to day 0 of the experiment (temperature: *P*-value < 0.001; RH: *P*-value < 0.001; α = 0.05). Finally, significant differences in PAR were also detected when we measured it at day 34 (*P* = 0.009; α = 0.05), following the rehydration period (Table S3), compared to day 0.

#### Physiological response

**Fig 2:**
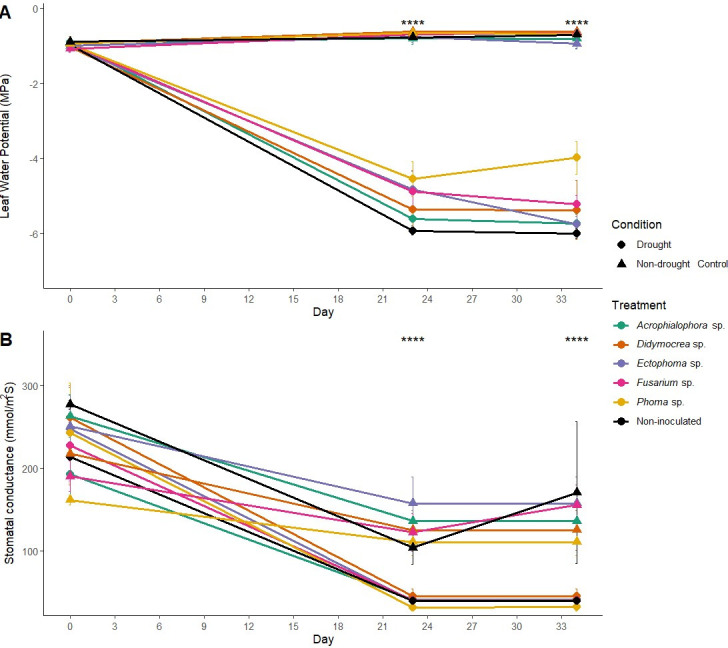
Physiological responses of *Theobroma cacao* ICS95 juvenile plants inoculated with fungal endophytes and non-inoculated plants subjected to drought conditions and non-drought control. Day 0 represents non-inoculated plants under normal conditions. Day 23 corresponds to plants inoculated and subjected to 14 days of drought stress. Day 34 shows plants after two weeks of rewatering. (**A**) Mean leaf water potential (Ψ_L_) with error bars. (**B**) Mean stomatal conductance (*K*_*s*_) of juvenile plants with error bars. The black lines indicate the mean Ψ_L_ or *K*_*s*_ of the treatment under non-inoculated control conditions. Asterisks denote statistical significance (****P < 0.0001, ***P < 0.001, **P < 0.01, and *P < 0.05) between drought vs non-drought control conditions according to Mann–Whitney test.

Leaf water potential and stomatal conductance were comparable across all treatments at the beginning of the experiment (day 0), showing no significant differences (Ψ_L_: *P*-value = 0.845, α = 0.05; *K*_*s*_: *P*-value = 0.217, α = 0.05; Fig. 2). By day 23, juvenile plants subjected to drought stress exhibited a significant reduction in both Ψ_L_ and *K*_*s*_ compared to those under non-drought conditions (*P*-value < 0.001, α = 0.05; Table 1 and Fig. 2).

**TABLE 1:** Mean leaf water potential (Ψ_L_, MPa) and stomatal conductance (*K*_*s*_, mmol/m^2^s) with standard deviation for the treatments at the beginning (day 0), after 14 days under drought conditions (day 23), and after re-watering the juvenile plants for 2 weeks (day 34)^*a*^

Treatment	Day 0		Day 23		Day 34	
	Ψ_L_	*K*_*s*_	Ψ_L_	*K*_*s*_	Ψ_L_	*K*_*s*_
*Ectophoma sp*.	−0.92 ±0.11	262.97 ±45.65	−5.6 ± 0.4	40.73 ± 11.42	−5.73 ± 0.45	40.73 ± 11.42
*Acrophialophora* sp.	−1.02 ± 0.25	250.7 ± 64.76	−4.82 ± 1.06	39 ± 17.91	−5.74 ± 0.36	39 ± 17.91
*Fusarium* sp.	−0.96 ± 0.18	189.9 ± 32.22	−4.88 ± 1.28	39.77 ± 7.65	−5.22 ± 0.41	39.77 ± 7.65
*Phoma* sp.	−0.96 ± 0.2	161.53 ± 10.22	−4.55 ± 1.03	31.32 ± 6.85	−3.98 ± 0.74*	32.17 ± 8.13
*Didymocrea* sp.	−1.03 ± 0.15	217.6 ± 66.02	−5.36 ± 1.07	44.87 ± 15.72	−5.37 ± 1.37	44.87 ± 15.72
Non-inoculated	−0.97 ± 0.17	213.37 ± 58.93	−5.92 ± 0.23	39.2 ± 11.86	−5.99 ± 0.24	39.2 ± 11.86

^*a*^ Statistical differences between treatments under drought conditions are represented by* (*P* ≤ 0.05).

After 14 days of drought stress (day 23), Kruskal-Wallis analysis revealed no statistically significant differences in Ψ_L_ among treatments under the drought condition (*H*-statistic = 7.42, *P* > 0.05, α = 0.05). However, juveniles inoculated with *Phoma* sp. exhibited significantly less negative Ψ_L_ values compared to non-inoculated plants, approaching values similar to the non-drought control (*Z*-statistic = 2.33, *P* = 0.02, α = 0.05). These analyses highlight the differential responses among fungal endophyte treatments (Fig. 2).

Under non-drought control conditions, *K*_*s*_ decreased after 23 and 34 days of the experiment compared to day 0 (*P*-value = 0.0025, α = 0.05). Juvenile plants inoculated with the five fungal endophytes experienced a decline in *K*_*s*_ on day 23, which stabilized by day 34. Meanwhile, non-inoculated juveniles showed a decrease in *K*_*s*_ until day 23 after 14 days of drought, followed by an increase on day 34 (*P*-value = 0.0927, α = 0.05) (Fig. 2).

At day 34, 2 weeks after re-watering, most juvenile plants failed to recover from drought stress. All plants inoculated with *Acrophialophora* sp. and non-inoculated controls previously exposed to drought died by the end of the experiment, exhibiting extremely negative Ψ_L_ values (≈ −6.0 MPa). Similarly, despite showing new leaf formation, most individuals inoculated with *Didymocrea* sp., *Ectophoma* sp., and *Fusarium* sp. maintained Ψ_L_ values near −6.0 MPa. In contrast, the two surviving *Phoma* sp. inoculated plants showed evidence of physiological recovery, with Ψ_L_ = −3.98 MPa, significantly less negative than other drought-stressed treatments. This value remained more negative than non-drought controls (Ψ_L_ = −0.69 MPa) but represented a recovery compared to mortality-level water potentials observed in other treatments. *Post-hoc* Dunn tests showed that *Phoma* sp. had significantly higher Ψ_L_ compared to non-inoculated plants at day 34 (*Z*-statistic = 2.72, *P* = 0.0066, α = 0.05; Fig. 2).

#### Plant growth

Regarding plant height, no significant differences were observed among treatments throughout the experiment (*P*-value = 0.5457, α = 0.05). However, significant variations (*P*-value = 0.0012, α = 0.05) were noted in the number of leaves between juvenile *T. cacao* plants subjected to drought stress and those in non-drought conditions. Specifically, drought-stressed plants exhibited a significant reduction in leaf count and SLA (Table S4) (*P*-value < 0.001, α = 0.05). In contrast, among plants under non-drought conditions, those inoculated with the fungus *Fusarium* sp. showed a significant increase in SLA (*P*-value < 0.001, α = 0.05) at both 23 and 34 days compared to non-inoculated plants and baseline measurements (Table S4).

Lastly, juveniles under drought conditions showed a significant decrease in the area of new leaves compared to the non-drought conditions control group (*P*-value < 0.001, α = 0.05). In contrast, juveniles inoculated with *Phoma* sp. showed a non-significant trend toward increased leaf area compared to controls at day 23 (Z-statistic = −0.665, *P*.adj = 0.505; Fig. S2). Similarly, *Fusarium* sp. showed a non-significant increase compared to *Phoma* sp. at day 34 (*Z*-statistic = 1.643, *P*.adj = 0.100; Fig. S3). Moreover, under non-drought conditions, the juveniles inoculated with *Ectophoma* sp. exhibited a significant (*Z*-statistic = −2.042, *P*-value = 0.0411, α = 0.05) increase in the area of the new leaves in comparison with the non-inoculated juveniles (Fig. S4).

**Fig 3:**
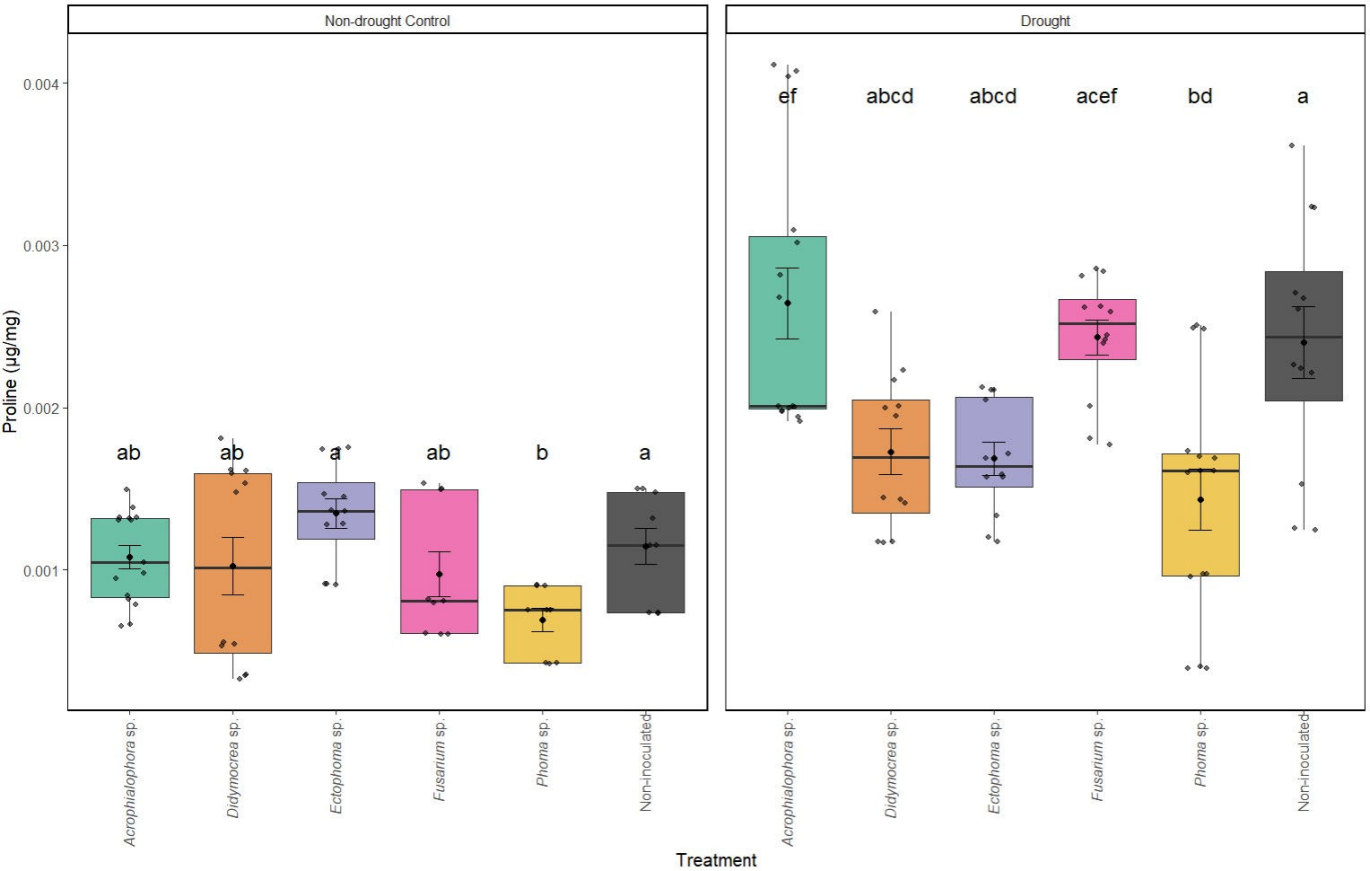
Free proline content (μg/mg) in leaves of juvenile plants of *Theobroma cacao* ICS95 after 14 days of non-drought control (left) and drought (right). Letters denote statistical significance using the Kruskal–Wallis test followed by Dunn’s *post hoc* test for the non-drought control, and ANOVA followed by Tukey’s test for the drought condition (*P* < 0.05).

On the other hand, drought-exposed juveniles showed significantly reduced RGR (*P* = 0.009, α = 0.05) compared to non-drought controls at day 34. Furthermore, under drought conditions, juveniles inoculated with *Ectophoma* sp. and *Fusarium* sp. exhibited less RGR reduction than non-inoculated plants, though these differences were not statistically significant (*P*-value = 0.998, α = 0.05).

#### Proline content

At day 0, no significant differences in proline content were observed between treatments. However, after 14 days of drought stress (day 23), a significant increase in leaf proline content was observed compared to those under non-drought control conditions (*P*-value < 0.001, α = 0.05). This increase in proline content, indicative of drought tolerance, was particularly pronounced in plants inoculated with *Acrophialophora* sp., *Fusarium* sp. 1, and in the non-inoculated group (Fig. 3). In contrast, treatments with *Phoma* sp., *Ectophoma* sp., and *Didymocrea* sp. exhibited a significantly smaller increase in proline levels compared to the non-inoculated group (*F*-statistic = 8.274, *P*-value < 0.001, α = 0.05). Interestingly, proline accumulation was significantly lower in plants inoculated with *Phoma* sp. and similar to those not subjected to drought.

## DISCUSSION

This study showed a new protocol to select fungal endophytes with a potential role to alleviate drought stress in plants. It also represents the first report on isolating and evaluating fungal endophytes from *Stenocereus* spp. in relation to their effects on *Theobroma cacao*. Our findings suggest that some fungal endophytes, such as *Phoma* sp., could enhance drought stress alleviation in juvenile *T. cacao* while simultaneously increasing the production of new leaves. This highlights the potential applications of these microorganisms to enhance agricultural resilience and sustainability and provide farmers with accessible microbial-based solutions for drought stress mitigation.

The endophytes *Fusarium* sp. and *Phoma* sp. isolated from *Stenocereus* spp. promoted plant growth under non-drought conditions and improved drought tolerance in juvenile plants of *T. cacao* ICS95, respectively. *Phoma* sp. even contributed to plant recovery after drought stress. According to Yarzábal Rodríguez et al. (48), extremotolerant fungi may exhibit an enhanced ability to confer drought tolerance in crops. Cacti roots represent a promising niche for discovering fungi that may enhance plant resilience. Some genera identified in this study, including *Alternaria* spp., *Aspergillus* spp., and *Fusarium* spp., are among the most frequently isolated fungi from plants in the Cactaceae family (49, 50). Additionally, records of *Diaporthe* spp. and *Rhodotorula* spp. have been noted in the study of Ferreira-Silva et al. (51) on endophytic fungi of *Melocactus ernestii*. Our study is the first to report *Polyporus* spp. as an endophyte in cacti, although this fungus was previously identified as capable of growing in xerophytic environments (52).

We isolated a diverse collection of fungal endophytes from the roots of cacti in both localities. Diversity of microbial communities may be shaped by several soil characteristics, particularly aridity (19) and pH (53). Similarly, Loro et al. (50) suggested that the diversity of endophytic fungi isolated from arid environments can be influenced by factors such as the type of isolation method that was used and the sample size. In this study, the main factors that could influence fungal diversity levels are the isolation protocol employed and the pH levels of the sampled environments. The reported electrical conductivities of Tatacoa and Taganga classify them as non-saline, suggesting that microbial respiration is not inhibited. However, the pH values were higher than eight in both locations, indicating that the soils are alkaline and characteristic of arid or semi-arid environments (53). Microorganisms growing in soils with high pH values can be considered either alkaliphilic or alkalitolerant (53). It is important to note that the diversity observed at each location may be underestimated, as it was determined solely based on culturable endophytic fungi isolated using a single growth medium (PDA). To enhance the understanding of fungal diversity present at each locality, complementary metabarcoding or metagenomic studies are recommended, alongside the use of diverse culture media and techniques that facilitate or optimize the isolation of xerophilic and alkaliphilic fungi (54).

Several endophytes, including *Acrophialophora* sp., *Didymocrea* sp., *Fusarium* sp., and *Phoma* sp., showed the ability to grow *in vitro* under intense drought stress. Interestingly, these genera have been previously reported as microorganisms with the potential to induce drought tolerance in various crops (55–58). Additionally, it has been reported that *Acrophialophora* sp., *Didymocrea* sp., *Phoma* sp., and *Fusarium* sp. may play a significant role in protection against plant pathogens, as well as exhibiting potential activity as growth promoters (55, 57–59).

Three approaches were employed to assess endophytic fungi’s role in inducing drought tolerance in *T. cacao*: physiological responses, relative growth rates, and biochemical responses to water stress. In the first approach, it was observed that juveniles of *T. cacao* ICS95 exposed to drought conditions reached Ψ_L_ values below −5.0 MPa, maintaining them above the level of fatal failure. Seleiman et al. (60) reported that values below −6.0 MPa can result in fatal hydraulic failures in plants. On the other hand, it is worth noting that the Ψ_L_ values obtained in this study are lower than those reported by Osorio-Zambrano et al. (1) for *T. cacao*, who found values of −3.0 and −3.5 MPa in juveniles under drought conditions. This discrepancy may be attributed to the fact that measurements in the Osorio-Zambrano et al. study (1) were taken during predawn hours, corresponding to more favorable conditions. In contrast, our measurements were recorded between 11:00 and 13:00, which is associated with more stressful plant conditions. Moreover, the experiment conducted by Osorio-Zambrano et al. (1) lasted for 26 days, in contrast to the 14 days of water stress applied in this study, after which the plants began to experience decay and wilting. Additionally, difference in results could be attributed to the microclimatic conditions under which both experiments were conducted and the amount of substrate used. In this study, the substrate for juveniles was 2 kg, while Osorio-Zambrano et al. used 5 kg (1). Less substrate means less water retention, reducing plant water availability (61).

*Theobroma cacao* ICS95 juveniles subjected to drought conditions significantly decreased Ψ_L_ compared to those under optimal conditions. This phenomenon is attributed to the reduction of available water in the soil, which causes an increase in the concentration of solutes within the plant (62, 63). As a defense mechanism, individuals close their stomata to minimize water loss, which explains the significant decrease in *K*_*s*_ compared to the control group (62, 64). However, stomatal closure reduces photosynthetic rate, which can compromise plant growth and yield (65).

Some fungal strains showed promising results, suggesting their potential use in enhancing various physiological parameters. Juveniles inoculated with *Phoma* sp. recovered better after drought stress, as indicated by significantly higher Ψ_L_ following the rewatering period. This phenomenon can be attributed to improved regulation of stomatal opening and reduced water loss through transpiration during the recovery phase (62, 64). The inoculated juveniles can maintain a more favorable osmotic balance, characterized by a lower solute concentration and higher water content, compared to non-inoculated plants or those inoculated with other fungi (66). In addition, a previous study showed that *Phoma* sp., when inoculated on *Pinus tabulaeformis*, can induce drought tolerance by significantly enhancing antioxidant activity (58).

A decrease in *K*_*s*_ was observed on days 23 and 34 in the non-drought control plants compared to day 0. This decline may be attributed to the significant temperature increase during this period. Elevated temperatures can trigger stomatal closure as a protective mechanism to reduce water loss and prevent the accumulation of ROS (67). It is important to note that, although stomatal closure occurred, the observed values remained within the optimal range reported for *T. cacao* (68).

Proline is an amino acid produced in response to water stress. It plays several critical roles in plants, including protection, maintaining osmotic balance, stabilizing membranes, and eliminating ROS (9, 56). Proline accumulates in plants in response to abiotic stressors, such as drought, protecting against these stresses (56, 69). Proline acts as a protective mechanism by stabilizing the membrane and neutralizing ROS, which affects the plant’s water potential (70, 71). Our results showed that *T. cacao* ICS95 juveniles exposed to drought conditions experienced a significant increase in proline production compared to non-drought control plants. The highest concentrations of proline were observed in non-inoculated juveniles and those inoculated with *Acrophialophora* sp. under drought conditions. Previous reports have shown higher abundances of this genus in saline soils (72) and tropical soils, and it is considered a thermotolerant genus (59). However, to the best of our knowledge, it has not been reported to affect proline content in plants under drought stress.

In contrast, plants inoculated with *Phoma* sp., *Ectophoma* sp., and *Didymocrea* sp. exposed to drought exhibited significantly smaller increases in proline levels than those under normal conditions. This result is intriguing because, according to Zhou et al. (58), *Phoma* spp. can increase proline levels in plants such as *Pinus tabulaeformis* under drought conditions. This increase is associated with the modulation of abscisic acid (ABA) signaling pathways, as ABA concentrations rise in response to low water availability. Elevated ABA levels promote the production of molecules associated with the induction of closing stomata as a protective response to water stress (73). Such a discrepancy was also observed with arbuscular mycorrhizal (AM) fungi (74). While drought stress induced an accumulation of proline in several AM plants, in other species, the concentration of proline in shoots was lower (74). The authors concluded that AM plants are less affected by water stress, resulting in lower proline levels due to the simultaneous inhibition of their synthesis and enhancement of their degradation (74). More studies are needed to determine if a similar phenomenon occurs in our research when cacao plants are inoculated with *Phoma* spp. and other genera that showed lower proline levels. A key finding for future studies is that cacao juveniles inoculated with *Phoma* sp. also exhibited lower proline accumulation in normal, non-drought conditions, suggesting that the fungus alters the proline metabolism.

Drought is considered one of the most significant abiotic stresses affecting the productivity and growth of *T. cacao* (75). According to Verbraeken et al. (76), plants experience a reduction in growth in response to water stress, as the limited availability of water hinders nutrient absorption, which is essential for plant development. Additionally, the loss of cellular turgor further compromises critical cellular processes required for growth (60, 77). These factors help explain the significant reduction in RGR observed in both inoculated and non-inoculated juvenile plants exposed to drought compared to those grown under non-drought stress conditions.

Finally, it has been reported that endophytic fungi can promote plant growth and increase leaf area through mechanisms such as the stimulation of phytohormones (78). This could explain the increase in leaf area and RGR observed in *T. cacao* juveniles inoculated with *Phoma* sp., previously reported as a growth promoter and the significant SLA with *Fusarium* sp. Nonetheless, care must be taken with extremophilic fungi that can adapt to higher temperatures and show undesirable pathogenicity (48). Additionally, specific genera isolated in this study have previously been reported as plant pathogens. Therefore, the eventual deployment of these strains in crops should be complemented by a pathogenic host range study.

This study provides a strategy to select fungi with the potential to alleviate drought stress in plants and the first comprehensive analysis of endophytic fungi associated with the cactus *Stenocereus* spp. Our findings reveal that both Tatacoa and Taganga display a wide variety of root-associated fungal species and complex community structures essential for maintaining ecosystem stability and resilience. This work brings valuable insights to developing biotechnological strategies to mitigate the adverse effects of a changing climate, particularly in drought-affected regions. The results support using fungal endophytes as a promising tool for enhancing agricultural resilience and sustainability in increasingly variable environmental conditions.

## SUPPLEMENTAL MATERIAL

Supplemental Material msphere.00865-25-s0001.pdf Tables S1 to S4; Fig. S1 to S4.

ASM does not own the copyrights to Supplemental Material that may be linked to, or accessed through, an article. The authors have granted ASM a non-exclusive, world-wide license to publish the Supplemental Material files. Please contact the corresponding author directly for reuse.
