# Supplementary material for: Fungal endophytes of cactus (Stenocereus spp.) as a potential alternative to alleviate drought stress in juveniles of Theobroma cacao L. ICS95
Source: mSphere. 2026 Apr 13;11(5):e00865-25. doi: 10.1128/msphere.00865-25 (PMC13203983; doi:10.1128/msphere.00865-25)

Supplementary Material

Supplementary Table 1

Supplementary Table 1. Geographic information of localities where the roots of *Stenocereus* spp. were sampled.

Department	Locality	Sample	Elevation (m.a.s.l)	Latitude	Longitude
Huila	Tatacoa	1	489	3.227898	-75.150175
Huila	Tatacoa	2	469	3.228118	-75.15031
Huila	Tatacoa	3	470	3.228342	-75.150432
Huila	Tatacoa	4	469	3.228132	-75.149673
Huila	Tatacoa	5	473	3.228327	-75.149527
Huila	Tatacoa	6	466	3.228808	-75.150205
Huila	Tatacoa	7	466	3.228785	-75.150878
Huila	Tatacoa	8	466	3.229205	-75.1512
Huila	Tatacoa	9	469	3.228932	-75.150497
Huila	Tatacoa	10	466	3.228808	-75.150205
Huila	Tatacoa	11	466	3.229547	-75.150115
Huila	Tatacoa	12	464	3.229375	-75.149462
Magdalena	Taganga	1	19	11.268545	-74.194403
Magdalena	Taganga	2	18	11.268445	-74.194498
Magdalena	Taganga	3	21	11.26837	-74.194575
Magdalena	Taganga	4	32	11.26854	-74.194612
Magdalena	Taganga	5	29	11.268662	-74.194668
Magdalena	Taganga	6	34	11.268818	-74.19467
Magdalena	Taganga	7	33	11.268642	-74.194708
Magdalena	Taganga	8	21	11.268735	-74.195045
Magdalena	Taganga	9	14	11.259083	-74.194953
Magdalena	Taganga	10	14	11.26913	-74.195888
Magdalena	Taganga	11	30	11.26905	-74.196407

Magdalena	Taganga	12	24	11.269178	-74.19608

Supplementary Table 2. Morphological and molecular identification of the fungal isolates.

**Best Blastn Result with ITS primers**	***Best Blastn Result with TEF primers***	**NCBI ID**	**Microscopic description**	**Macroscopic descrption**	**Morphology Identification**	**Locality**
Acrocalymma sp.		PV938402	No data available.	Velvety colony with white to grey color (approx. RGB 255, 242, 206)	No data available.	Tatacoa (Huila)
Acrophialophora sp.		PX218687	Dark, thick, septate mycelium. Bottle-shaped phialides producing hyaline, ellipsoidal ameroconidia. Pycnidia formation observed.	Colony with leathery to velvety texture and grey color (approx. RGB 0, 72, 66). White ring at the outer margin (approx. RGB 254, 249, 255)	*Acrophialophora* sp.	Taganga (Magdalena)
Alternaria sp.		PV938419	Mycelium is dematiaceous, thick-walled, and septate. Dictyoconidia dematiaceous produced in acropetal chains.	Velvety brown colony in the obverse and reverse (approx. RGB 134, 93, 0).	*Alternaria* sp.	Tatacoa (Huila) - Taganga (Magdalena)

Aspergillus arcoverdensis		PV938425	Hyaline, thin mycelium. Conidiophores with terminal vesicles bearing bottle-shaped phialides, from which acropetal chains of hyaline, ovoid conidia arise.	Colony granular with green color in the obverse (approx. RGB 134, 188, 0) and white in the reverse (approx. RGB 252, 255, 226).	*Aspergillus* sp.	Tatacoa (Huila) - Taganga (Magdalena)
Aspergillus eucalypticola		PV938389	Hyaline, thin mycelium. Conidiophores with terminal vesicles bearing bottle-shaped phialides, from which acropetal chains of hyaline, ovoid conidia arise.	Colony with a granular texture, black on the surface (approx. RGB 0, 41, 4) and white (approx. RGB 252, 255, 226) on the reverse side.	*Aspergillus* sp.	Tatacoa (Huila) - Taganga (Magdalena)
Aspergillus flavus		PV938412	Hyaline, thin mycelium. Conidiophores with terminal vesicles bearing bottle-shaped phialides, from which acropetal chains of hyaline, ovoid conidia arise.	Colony with granular texture, green to yellow color (approx. RGB 203, 200, 3) in the obverse and grey in the reverse (approx. RGB 0, 84, 85).	*Aspergillus* sp.	Tatacoa (Huila) - Taganga (Magdalena)

Aspergillus heldtiae		PV938408	Hyaline, thin mycelium. Conidiophores with terminal vesicles bearing bottle-shaped phialides, from which acropetal chains of hyaline, ovoid conidia arise.	Colony with granular texture, yellow (approx. RGB 254, 232, 38) color in the obverse and yellow-green (approx. RGB 196, 213, 0) in the reverse.	*Aspergillus* sp.	Tatacoa (Huila) - Taganga (Magdalena)
Aspergillus lentulus		PV938426	Hyaline, thin mycelium. Conidiophores with terminal vesicles bearing bottle-shaped phialides, from which acropetal chains of hyaline, ovoid conidia arise.	Velvety colony white with pink in the center (approx. RGB 250, 237, 255).	*Aspergillus* sp.	Tatacoa (Huila) - Taganga (Magdalena)
Aspergillus neoterreus		PV938403	Hyaline, thin mycelium. Conidiophores with terminal vesicles bearing bottle-shaped phialides, from which acropetal chains of hyaline, ovoid conidia arise.	Colony with a granular texture, black on the surface (approx. RGB 0, 70, 70) and white (approx. RGB 255, 239, 229) on the reverse side.	*Aspergillus* sp.	Tatacoa (Huila) - Taganga (Magdalena)

Aspergillus niger		PV938420	Hyaline, thin mycelium. Conidiophores with terminal vesicles bearing bottle-shaped phialides, from which acropetal chains of hyaline, ovoid conidia arise.	Colony granular with black color (approx. RGB 2, 26, 0) in the obverse and white (approx. RGB 248, 255, 250) in the reverse.	*Aspergillus* sp.	Tatacoa (Huila) - Taganga (Magdalena)
Aspergillus terreus		PV938418	Hyaline, thin mycelium. Conidiophores with terminal vesicles bearing bottle-shaped phialides, from which acropetal chains of hyaline, ovoid conidia arise.	Colony granular with yellow color (approx. RGB 245, 215, 41) in the obverse and brown (approx. RGB 178, 118, 1) in the reverse. In some cases, yellow synnemata are formed.	*Aspergillus* sp.	Tatacoa (Huila) - Taganga (Magdalena)
Aureobasidium sp.		PV938395	No data available.	Velvety white to grey (approx. RGB 244, 230, 255) in the obverse and grey (approx. RGB 1, 68, 86) in the reverse.	No data available.	Tatacoa (Huila) - Taganga (Magdalena)
Clavulina sp.		PV938405	No data available.	Velvety colony with grey to pink color (approx. RGB 254, 221, 237).	No data available.	Tatacoa (Huila)

Coprinellus sp.		PV938409	No data available.	Velvety colony with white color (approx. RGB 246, 254, 243) in the obverse and pink to brown (approx. RGB255, 192, 203) in the reverse.	No data available.	Tatacoa (Huila)
Curvularia alcornii		PV938410	Mycelium is dematiaceous, thick-walled, and septate. Fragmoconidia dematiaceous produced from sympodial conidiogenous cells.	Velvety colony with grey color (approx. RGB 0, 92, 71) in the obverse and black color (approx. RGB 217, 30, 0) in the reverse.	*Curvularia* sp.	Tatacoa (Huila) - Taganga (Magdalena)
Curvularia buchloes		PV938401	Mycelium is dematiaceous, thick-walled, and septate. Fragmoconidia dematiaceous produced from sympodial conidiogenous cells.	Velvety grey to black (approx. RGB 1, 14, 58) in the obverse and in the reverse	*Curvularia* sp.	Tatacoa (Huila) - Taganga (Magdalena)
Curvularia coimbatorensis		PV938393	Mycelium is dematiaceous, thick-walled, and septate. Fragmoconidia dematiaceous produced from sympodial conidiogenous cells.	Velvety colony with grey color (approx. RGB 0, 92, 71). The reverse was black (approx. RGB 217, 30, 0).	*Curvularia* sp.	Tatacoa (Huila) - Taganga (Magdalena)

Curvularia milisiae		PV938407	Mycelium is dematiaceous, thick-walled, and septate. Fragmoconidia dematiaceous produced from sympodial conidiogenous cells.	Velvety black (approx. RGB 0, 27, 24) in the obverse and in the reverse.	*Curvularia* sp.	Tatacoa (Huila) - Taganga (Magdalena)
Curvularia pseudolunata		PV938394	Mycelium is dematiaceous, thick-walled, and septate. Fragmoconidia dematiaceous produced from sympodial conidiogenous cells.	Velvety colony brown (approx. RGB 78, 64, 0) with orange lines (approx. RGB 174, 95, 0) and reverse orange (approx. RGB 174, 95, 0) with black lines (approx. RGB 44, 34, 0).	*Curvularia* sp.	Tatacoa (Huila) - Taganga (Magdalena)
Curvularia simmonsii		PV938400	Mycelium is dematiaceous, thick-walled, and septate. Fragmoconidia dematiaceous produced from sympodial conidiogenous cells.	Velvety colony grey (approx. RGB 30, 46, 1) in the obverse and in the reverse.	*Curvularia* sp.	Tatacoa (Huila) - Taganga (Magdalena)
Deniquelata sp.		PV938427	Mycelium is hyaline, thin, and sparsely septate. Subglobose conidia produces by conidiogenous cells subcylindrical.	Velvety colony white in the obverse and in the reverse (approx. RGB 246, 254, 231), with ring at the outer margin yellow (approx. RGB 255, 234, 166).	No data available.	Tatacoa (Huila)

Diaporthe sp.		PV938422	Mycelium is hyaline, thin, and sparsely septate. Ameroconidia fusiform.	Velvety-Cottony white (approx. RGB 246, 254, 231) colony in the obverse and in the reverse with ring at the outer margin black.	No data available.	Tatacoa (Huila)
Didymella sp.		PV938413	Mycelium is hyaline, thin, and sparsely septate. No reproductive structures were observed under the examined conditions.	Velvety grey (approx. RGB 30, 46, 1) colony in the obverse and in the reverse with ring at the outer margin white (approx. RGB 246, 254, 231).	No data available.	Tatacoa (Huila)
Didymella sp.		PV938424	Mycelium is hyaline, thin, and sparsely septate. No reproductive structures were observed under the examined conditions.	Colony cottony white (approx. RGB 246, 254, 231) in the obverse and in the reverse.	No data available.	Tatacoa (Huila)
Didymocrea sp.		PX218685	Dark, thick, and septate mycelium. Micronematous conidiophores producing solitary, dematiaceous conidia acrogenously.	Velvety colony with grey (approx. RGB 30, 46, 1) color in the obverse and black color in the reverse (approx. RGB 0, 8, 37).	*Didymocrea* sp.	Tatacoa (Huila)

Ectophoma sp.		PX218686	Dark, septate mycelium and produced brown pycnidia containing ellipsoidal ameroconidia. Each conidium presented two guttules, and chlamydospores were observed forming in chains	Velvety-leathery colony with grey (approx. RGB 30, 46, 1) color in the obverse and black color in the reverse (approx. RGB 0, 8, 37).	*Phoma* sp.	Tatacoa (Huila) - Taganga (Magdalena)
Epicoccum sp.		PV938396	No data available.	Colony with leathery texture and black color (approx. RGB 0, 8, 37).	No data available.	Tatacoa (Huila) - Taganga (Magdalena)
Fusarium falciforme	*Neocosmospora rubicola*	FEC28_15072025	Hyaline, thin, septate mycelium. Ameroconidia and hyaline macroconidia (didymoconidium), produced on flask-shaped phialides.	Cottony, ivory white in color (approx. RGB 254, 243, 213), with scarce aerial mycelium. The colony surface was smooth and uniform, with entire margins.	*Fusarium* sp.	Tatacoa (Huila) - Taganga (Magdalena)
Fusarium falciforme	*Neocosmospora* sp.	FEC30_15072025	Hyaline, thin, septate mycelium. Ameroconidia and hyaline macroconidia (didymoconidium), produced on flask-shaped phialides.	Cottony, ivory white in color (approx. RGB 254, 243, 213), with scarce aerial mycelium. The colony surface was smooth and uniform, with entire margins.	*Fusarium* sp.	Tatacoa (Huila) - Taganga (Magdalena)

Fusarium falciforme	*Neocosmospora* sp.	FEC31_15 072025	Hyaline, thin, septate mycelium. Ameroconidia and hyaline macroconidia (didymoconidium), produced on flask-shaped phialides.	Cottony, ivory white in color (approx. RGB 254, 243, 213), with scarce aerial mycelium. The colony surface was smooth and uniform, with entire margins.	*Fusarium* sp.	Tatacoa (Huila) - Taganga (Magdalena)
Fusarium sp.	*Fusarium* sp.	FEC29_15 072025	Hyaline, thin, septate mycelium. Hyaline didymoconidium micro and macroconida, produced on flask-shaped phialides.	Cottony, ivory white in color (approx. RGB 254, 243, 213), with scarce aerial mycelium. The colony surface was smooth and uniform, with entire margins.	No data available.	Tatacoa (Huila) - Taganga (Magdalena)
Lasiodiplodia sp.		PV938421	Mycelium is septate and hyaline. No reproduction structures	Colony cottony grey (approx. RGB 1, 22, 51) in the obverse and black in the reverse (approx. RGB 0, 8, 37).	*Lasiodiplodia* sp.	Tatacoa (Huila) - Taganga (Magdalena)
Latorua sp.		PV938391	Hyaline, thin, septate mycelium. Simple, geniculate, macronematous conidiophore. Ellipsoidal conidia in acropetal chains.	Velvety colony with grey color (approx. RGB 1, 22, 51) with ring at the outer margin. The reverse was grey.	*Acroconidiella* sp.	Taganga (Magdalena)
Macrophomina sp.		PV938415	No data available.	Velvety colony grey (approx. RGB 1, 22, 51) in the obverse and in the reverse.	*Macrophomina* sp.	Tatacoa (Huila) - Taganga (Magdalena)

Moniliophthora sp.		PV938414	No data available.	Velvety white (approx. RGB 1, 22, 51) in the obverse and in the reverse	No data available.	Tatacoa (Huila) - Taganga (Magdalena)
Monosporascus sp.		PV938392	No data available.	Cottony, ivory white in color (approx. RGB 242, 254, 232), with scarce aerial mycelium. The colony surface was smooth and uniform, with entire margins.	No data available.	Tatacoa (Huila) - Taganga (Magdalena)
Myrmaecium sp.		PV938423	No data available.	Velvety grey colony (approx. RGB 1, 22, 51) in the obverse and brown (approx. RGB 139, 117, 0) in the reverse.	No data available.	Taganga (Magdalena)
Oblongocollomyces zhivanae		PV938404	No data available.	Velvety colony with grey color (approx. RGB 20, 44, 66).	No data available.	Tatacoa (Huila)
Paraboeremia sp.		PV938398	No data available.	Colony with leathery texture and brown color (approx. RGB 94, 33, 1).	No data available.	Tatacoa (Huila)

Phoma sp.		PX218688	Mycelium is thick-walled and septate, ranging from hyaline to dematiaceous. Pycnidia are dark, globose to subglobose, and ostiolate. Conidia formed within pycnidia are ovoid, aseptate, and vary in pigmentation from hyaline to dematiaceous. Each conidium with two guttules.	Velvety colony with brown-yellow (approx. RGB 124, 116, 2) color in the obverse.	*Phoma* sp.	Tatacoa (Huila)
Poaceascoma sp.		PV938411	No data available.	Velvety colony with brown-grey color in the obverse and black color in the reverse (approx. RGB 87, 58, 0).	No data available.	Tatacoa (Huila) - Taganga (Magdalena)
Polyporus sp.		PV938390	Mycelium hyaline, thin, and sparsely septate. No reproductive structures were observed.	The colony was white (approx. RGB 254, 243, 213) with a yellow (approx RGB 255, 245, 196) ring at the outer margin and a velvety texture on the surface. The reverse was initially white (approx. RGB 254, 243, 213), becoming brown (approx. RGB 223,	No data available.	Taganga (Magdalena)

				168, 1) at the center over time.		
Purpureocillium lilacinum		PV938406	Hyaline, septate mycelium with erect conidiophores bearing flask-shaped phialides that produce chains of hyaline, ellipsoidal conidia.	Velvety colony with brown color (approx. RGB 97, 34, 1) in obverse and reverse. Production of yellow pigment (approx. RGB 193, 164, 1).	*Purpureocillium* sp.	Tatacoa (Huila)
Rhizopus sexualis		PV938399	Dark, thick, and sparsely septate mycelium. Presence of dematiaceous sporangia and sporangiophores, along with well-developed rhizoids.	Cottony colony white-grey (approx. RGB 237, 245, 254) in the obverse and grey in the reverse.	*Rhizopus* sp.	Tatacoa (Huila) - Taganga (Magdalena)
Rhodotorula sp.		PV938417	Cells are unicellular, globose to ovoid, and reproduce by multilateral budding.	Colonies are smooth, moist to mucoid, and display a characteristic pink color (approx. RGB 255, 174, 237).	*Rhodotorula* sp.	Taganga (Magdalena)

Trichoderma saturnisporum		PV938397	Septate, hyaline mycelium with branched conidiophores bearing flask-shaped phialides that produce blue ameroconidia.	Cottony texture with white color (approx. RGB 236, 255, 230) with cooncentric green-blue rings (approx. RGB 1, 138, 58).	*Trichoderma* sp.	Tatacoa (Huila) - Taganga (Magdalena)
Trichoderma sp.		PV938416	Septate, hyaline mycelium with branched conidiophores bearing flask-shaped phialides that produce green ameroconidia.	Colony cottony white (approx. RGB 253, 255, 245) in the obverse and in the reverse.	*Trichoderma* sp.	Taganga (Magdalena)
	Cylindrocarpon sp.	FEC32_15072025	Hyaline, thin, septate mycelium. Hyaline didymoconidium micro and macroconida, produced on flask-shaped phialides.	Cottony, ivory white in color (approx. RGB 254, 243, 213), with scarce aerial mycelium. The colony surface was smooth and uniform, with entire margins.	*Fusarium* sp.	Tatacoa (Huila) - Taganga (Magdalena)
	Bisifusarium sp.	FEC49_15072025	Hyaline, thin, septate mycelium. Hyaline didymoconidium micro and macroconida, produced on flask-shaped phialides.	Cottony, ivory white in color (approx. RGB 251, 240, 254), with scarce aerial mycelium. The colony surface was smooth and uniform, with entire margins.	*Fusarium* sp.	Tatacoa (Huila) - Taganga (Magdalena)

Supplementary Table 3. Daily (7:00 am – 6:00 pm) average environmental conditions during the greenhouse assays.

Day	Temp min (°C)	Temp max (°C)	Relative Humidity (%)	PAR (μmol/m²/s)
0	25.761	26.347	78.205	151.825
1	29.077	29.436	62.788	198.057
2	26.954	27.855	72.030	199.369
3	25.441	25.451	76.420	139.619
4	25.961	26.321	83.968	149.615
5	22.893	23.376	87.558	95.832
6	26.686	28.082	69.225	176.771
7	26.204	27.632	71.108	138.057
8	27.621	28.969	64.929	161.164
9	27.189	27.662	67.797	153.593
10	24.043	23.848	79.496	111.599
11	27.320	27.113	66.622	150.593
12	26.751	25.753	70.279	164.129
13	29.910	29.337	62.469	203.410
14	28.271	26.824	82.817	141.446
15	29.620	28.957	69.213	190.838
16	24.632	24.638	81.351	104.141
17	28.549	27.039	70.960	172.271
18	30.398	29.641	70.382	185.486
19	27.704	27.549	68.912	170.475
20	26.805	27.358	76.499	147.722
21	24.513	24.564	82.115	112.245
22	28.847	29.650	63.477	183.700
23	26.278	25.800	77.169	143.664
24	28.867	29.420	61.424	181.057
25	27.735	27.802	63.734	180.950
26	24.470	23.524	80.566	103.590
27	24.860	24.196	74.991	100.729
28	25.903	25.159	73.913	142.649
29	27.201	26.400	67.026	181.414
30	25.297	25.224	68.897	151.307
31	22.771	23.121	74.516	91.526
32	22.108	21.989	77.358	77.359
33	23.814	23.950	71.901	93.075
34	22.248	22.485	81.573	97.200

Supplementary Table 4. Means of morphological parameters of juveniles of Theobroma cacao ICS95 exposed under drought and normal conditions, measured on different days of the experiment.

Day	Treatment	Conditions	N° Leaves	Height (cm)	Leaf area (cm^2)	Leaf fresh weight (g)	Stem fresh weight (g)	Root fresh weight (g)	Leaf dry weight (g)	Stem dry weight (g)	Root dry weight (g)	Biomass (g)	SLA
0	*Ectophoma* sp.	Drought	12.33 ± 3.06	38.17 ± 2.02	37.93 ± 2.36	4.6 ± 1.59	16.43 ± 1.62	7.47 ± 0.76	1.15 ± 0.27	4.12 ± 0.41	2.23 ± 0.74	7.5 ± 1.26	349.71 ± 130.87
0	*Ectophoma* sp.	Non-drought Control	14.33 ± 1.15	36.7 ± 2.65	28.24 ± 3.15	4.83 ± 0.15	13.87 ± 0.06	5.4 ± 1.13	1.2 ± 0.27	2.93 ± 0.87	1.39 ± 0.26	5.53 ± 1.15	456.33 ± 552.69
0	*Acrophialophora* sp.	Drought	11 ± 1	37.17 ± 2.57	27.55 ± 6.98	4.67 ± 1.36	9.73 ± 3.75	4.93 ± 1.89	1.38 ± 0.53	2.4 ± 0.47	1.54 ± 0.77	5.33 ± 0.78	558.06 ± 355.92
0	*Acrophialophora* sp.	Non-drought Control	13 ± 1	36.5 ± 3.04	24.79 ± 5.05	4.63 ± 0.93	11.6 ± 0.53	7.77 ± 3.93	1.28 ± 0.38	3.06 ± 0.35	1.7 ± 0.46	6.04 ± 0.9	272.76 ± 209.56
0	*Didymocrea* sp.	Drought	12.67 ± 2.31	37.37 ± 1.31	37.3 ± 2.89	3.93 ± 0.21	10.6 ± 3.12	4.73 ± 2.02	1.06 ± 0.18	2.79 ± 1.12	1.24 ± 0.59	5.09 ± 1.84	548.67 ± 397.56
0	*Didymocrea* sp.	Non-drought Control	12 ± 1.73	37.93 ± 2.61	26.15 ± 7.87	3.67 ± 0.8	12.37 ± 1.83	6.1 ± 2.14	0.93 ± 0.18	2.66 ± 1.26	1.57 ± 0.56	5.16 ± 1.81	496.96 ± 497.64
0	*Phoma* sp.	Drought	11 ± 3	37.07 ± 1.1	34.23 ± 2.05	3.37 ± 0.59	13.44 ± 2.17	5.8 ± 1.01	0.88 ± 0.11	2.89 ± 0.57	1.71 ± 0.54	5.47 ± 0.78	336.21 ± 129.72
0	*Phoma* sp.	Non-drought Control	13.67 ± 2.08	37.5 ± 3	34.01 ± 10.28	3.9 ± 1.01	12.67 ± 1.81	4.3 ± 3.06	1.16 ± 0.25	2.77 ± 0.44	1.21 ± 0.83	5.13 ± 1.16	348.96 ± 142.47
0	*Fusarium* sp.1	Drought	10.33 ± 2.52	37.33 ± 2.02	28.75 ± 6.82	2.7 ± 0.53	12.97 ± 2.84	5.23 ± 1.64	0.71 ± 0.12	3.42 ± 0.58	1.4 ± 0.47	5.53 ± 1.17	461.05 ± 322.54
0	*Fusarium* sp.1	Non-drought Control	11.67 ± 0.58	36.33 ± 1.04	27.02 ± 5.76	2.73 ± 0.76	10.47 ± 1.1	4.3 ± 1.66	0.78 ± 0.2	2.68 ± 0.27	1.13 ± 0.54	4.6 ± 0.9	304.38 ± 200.68
0	Non-inoculated	Drought	14 ± 1	39.33 ± 2.99	33.74 ± 5.58	6.2 ± 1.91	13.37 ± 0.65	5.73 ± 1.12	1.75 ± 0.44	2.45 ± 0.76	1.41 ± 0.42	5.6 ± 0.55	376.17 ± 131.79
0	Non-inoculated	Non-drought Control	13.67 ± 0.58	36.83 ± 1.86	33.61 ± 4.59	5.23 ± 0.15	15.07 ± 4.21	7.33 ± 3.54	1.49 ± 0.12	3.74 ± 1.05	1.82 ± 0.96	7.06 ± 2.04	517.42 ± 469.77
23	*Ectophoma* sp.	Drought	11.6 ± 2.88	36.68 ± 1.53	7 ± 15.64	1.98 ± 0.83	7.12 ± 3.46	3.46 ± 1.58	1.26 ± 0.59	4.1 ± 1.57	1.41 ± 0.66	6.77 ± 2.16	233.05 ± 150.46
23	*Ectophoma* sp.	Non-drought Control	14.2 ± 4.15	36.84 ± 1.52	41.3 ± 19.23	5.24 ± 1.12	15.26 ± 1.73	9.5 ± 1.43	1.42 ± 0.46	4.46 ± 0.68	2.73 ± 0.6	8.61 ± 1.21	206.73 ± 61.06
23	*Acrophialophora* sp.	Drought	11.2 ± 3.63	35.5 ± 2.39	6.42 ± 9.51	1.58 ± 0.8	8.08 ± 2	3.96 ± 1.56	1.03 ± 0.46	3.56 ± 0.69	1.52 ± 0.66	6.12 ± 0.97	199.89 ± 30.5
23	*Acrophialophora* sp.	Non-drought Control	10.4 ± 3.36	38.22 ± 2.1	49.07 ± 19.99	6.14 ± 4.77	13.78 ± 1.81	9.6 ± 0.73	1.45 ± 0.86	3.96 ± 0.4	2.62 ± 0.49	8.03 ± 0.46	194.47 ± 16.81
23	*Didymocrea* sp.	Drought	11.4 ± 0.89	38.4 ± 2.95	5.07 ± 11.33	1.76 ± 0.33	7.86 ± 2.47	3.26 ± 0.38	1.19 ± 0.21	3.26 ± 1.74	1.73 ± 0.31	6.17 ± 1.71	233.43 ± 43.54
23	*Didymocrea* sp.	Non-drought Control	13.33 ± 1.53	36.73 ± 1.12	31.03 ± 8.27	5.23 ± 1.7	16.07 ± 3.57	9.33 ± 1.98	1.33 ± 0.4	4.63 ± 1.25	2.91 ± 0.85	8.86 ± 2.09	260.6 ± 80.6
23	*Phoma* sp.	Drought	13.6 ± 5.41	37.76 ± 1.21	16.22 ± 14.97	2.14 ± 0.79	7.82 ± 2.2	4.92 ± 3.28	1.3 ± 0.85	3.99 ± 1.3	2.24 ± 1.29	7.53 ± 2.15	210.74 ± 30.85
23	*Phoma* sp.	Non-drought Control	13.4 ± 1.34	38.1 ± 3.06	38.53 ± 10.3	4.76 ± 2.12	16.74 ± 3.69	9.64 ± 1.31	1.38 ± 0.4	4.74 ± 1.68	3.21 ± 1.32	9.32 ± 2.23	290.93 ± 125.49

23	*Fusarium* sp.1	Drought	10.8 ± 3.9	35.9 ± 4.16	6.82 ± 9.46	1.78 ± 0.95	7.04 ± 1.32	3.48 ± 0.85	1.15 ± 0.7	3.28 ± 0.92	1.89 ± 0.85	6.32 ± 1.91	215.61 ± 27.45
23	*Fusarium* sp.1	Non-drought Control	11.4 ± 3.29	38.86 ± 1.05	32.01 ± 4.19	4 ± 3	13.36 ± 6.56	9.22 ± 5.26	1.23 ± 0.75	3.93 ± 1.84	2.11 ± 0.93	7.27 ± 2.59	230.18± 14565.86
23	Non-inoculated	Drought	10.2 ± 3.27	38 ± 3.94	13.17 ± 19.04	2.18 ± 1.09	9.6 ± 2.67	5.72 ± 1.94	1.17 ± 0.51	4.33 ± 0.86	2.54 ± 0.98	8.04 ± 1.32	210.01 ± 51.16
23	Non-inoculated	Non-drought Control	14 ± 3.16	35.5 ± 2.29	34.7 ± 6.69	5.16 ± 1.93	13.44 ± 4.48	6.4 ± 4.2	1.66 ± 0.76	4.51 ± 1.51	2.99 ± 0.75	9.16 ± 1.68	217.18 ± 26.12
34	*Ectophoma* sp.	Drought	13.33 ± 2.08	36.67 ± 4.54	31.56 ± NA	1.5 ± NA	8.2 ± 3.08	9.87 ± 4.27	1.37 ± NA	5.12 ± NA	3.46 ± NA	3.32 ± 5.75	208.29 ± NA
34	*Ectophoma* sp.	Non-drought Control	16.67 ± 10.69	36 ± 2.18	61.26 ± 28.21	8.63 ± 5.28	14.07 ± 2.9	10.1 ± 2.4	2.1 ± 1.09	3.44 ± 0.59	2.8 ± 1.31	8.33 ± 0.72	187.81 ± 39.01
34	*Acrophialophora* sp.	Drought	13 ± 2.65	33.3 ± 1.57	---	---	4.83 ± 0.76	5.5 ± 0.46	---	---	---	---	---
34	*Acrophialophora* sp.	Non-drought Control	19 ± 3.61	35.5 ± 3.04	51.67 ± 16	8.7 ± 4.51	11.3 ± 3.38	7.67 ± 3.46	2.37 ± 0.93	2.94 ± 1.51	2.11 ± 1.1	7.42 ± 2.08	297.84 ± 30.17
34	*Didymocrea* sp.	Drought	12 ± 1.73	40.43 ± 5.58	31 ± NA	1.2 ± NA	7.17 ± 1.1	10.63 ± 6.03	1.14 ± NA	2.18 ± NA	1.3 ± NA	1.54 ± 2.66	210.84 ± NA
34	*Didymocrea* sp.	Non-drought Control	11.67 ± 0.58	35 ± 3.12	37.33 ± 8.15	4.83 ± 2.12	12.77 ± 1.55	10.83 ± 4.31	1.75 ± 0.77	4.19 ± 0.98	3.43 ± 1.48	9.37 ± 1.48	150.29 ± 31.55
34	*Phoma* sp.	Drought	7.67 ± 3.79	37 ± 1.73	29.03 ± NA	0.5 ± NA	6.73 ± 1.1	5.37 ± 2.16	0.14 ± NA	2.35 ± NA	0.99 ± NA	1.16 ± 2.01	189.58 ± NA
34	*Phoma* sp.	Non-drought Control	11.67 ± 2.52	38.67 ± 1.26	50.21 ± 9.77	5.23 ± 1.38	17.9 ± 6.6	15.1 ± 7.63	1.73 ± 0.18	6.52 ± 0.92	7.05 ± 2.14	15.3 ± 2.88	221.78 ± 44.36
34	*Fusarium* sp.1	Drought	11 ± 5.2	37.5 ± 4.27	40.58 ± NA	2.8 ± NA	8.1 ± 5.43	8.83 ± 3.56	2.36 ± NA	4.96 ± NA	5 ± NA	4.11 ± 7.12	---
34	*Fusarium* sp.1	Non-drought Control	18.33 ± 5.51	34.67 ± 0.76	35.21 ± 6.24	8.57 ± 1.68	12.8 ± 4.07	9.63 ± 5.78	2.5 ± 0.38	2.6 ± 0.52	2.06 ± 0.78	7.16 ± 1.15	225.23 ± 23.12
34	Non-inoculated	Drought	9 ± 3.46	37.43 ± 3.46	---	---	7.53 ± 3.03	9.43 ± 2.44	---	---	---	---	200.84 ± NA
34	Non-inoculated	Non-drought Control	13 ± 4.36	37.5 ± 1.32	50.31 ± 15.59	6.5 ± 2.25	19.63 ± 5.61	20.93 ± 2.48	2.23 ± 0.72	5.14 ± 0.16	4.17 ± 0.05	11.54 ± 0.85	209.35 ± 12.73

Supplementary Figure 1. Mean soil water content (SWC, m³/m³) over time, measured daily, with error bars indicating standard deviation. Green vertical line on day 6 indicates inoculation of the soil with the fungal endophytes. Red vertical line on day 9 indicates the onset of drought stress. Blue vertical line at day 23 indicates the end of drought stress and the beginning of the re-irrigation.

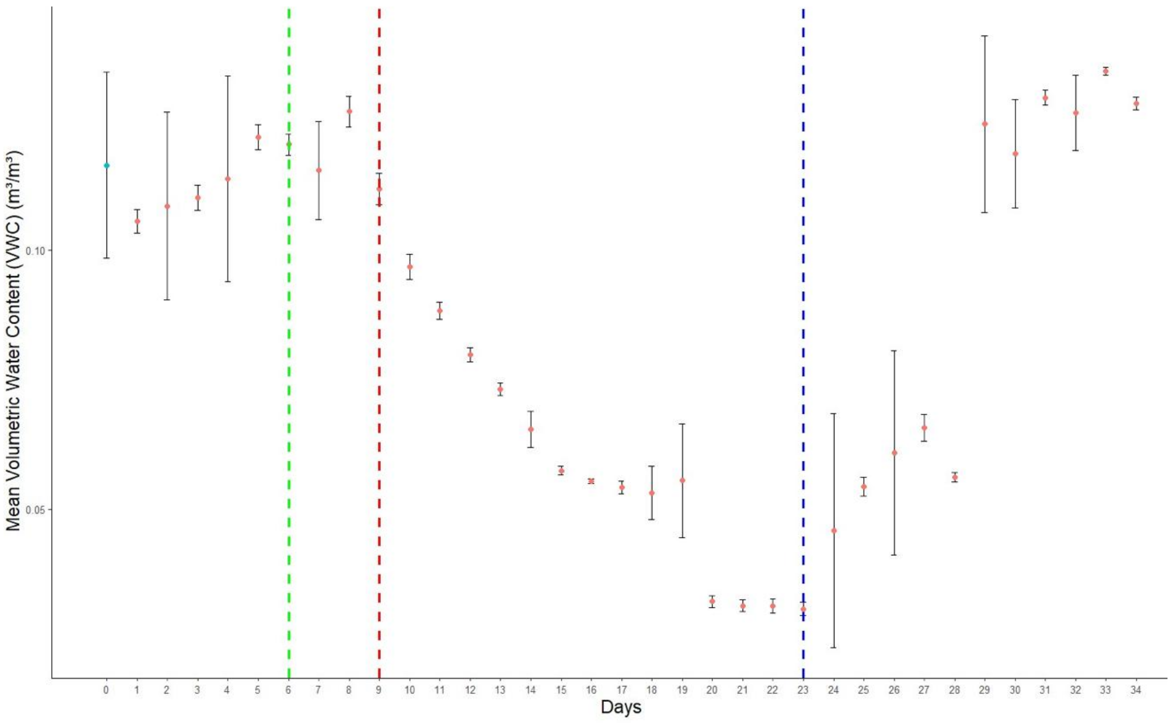

Supplementary Figure 2. Mean leaf area (cm²) of *Theobroma cacao* ICS95 juveniles exposed to non-drought control (left) and drought conditions for 14 days (right) (day 23). Letters indicate significant differences determined by Kruskal-Wallis with Dunn post hoc test for treatments under non-drought control, and drought conditions

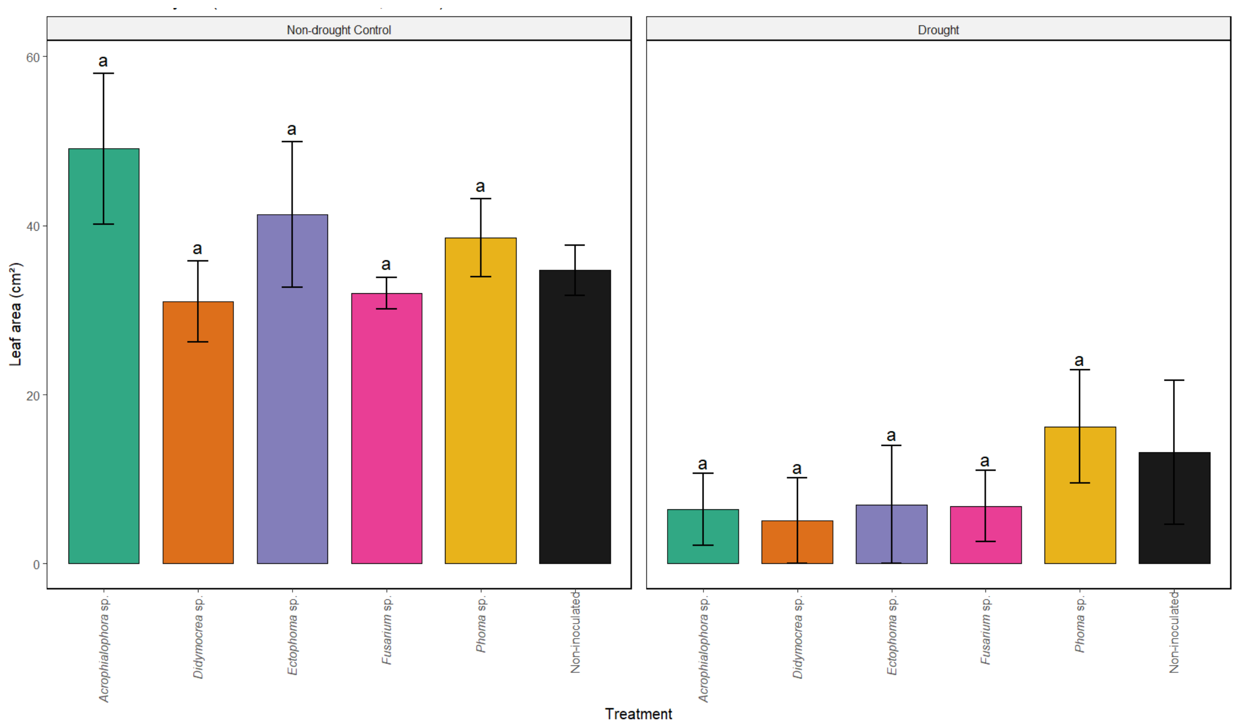

Non-drought Control
Drought
Leaf area (cm²)
60
40
20
0
a
Acrophialophora sp.
Didymocrea sp.
Ectophoma sp.
Fusarium sp.
Phoma sp.
Non-inoculated
Treatment

Supplementary Figure 3. Mean leaf area (cm²) of *Theobroma cacao* ICS95 juveniles exposed to non-drought control (left) and drought conditions followed by rewatering for two weeks (right), measured at the end of the recovery period. Error bars are not displayed due to low replication; only 1-2 plants survived per treatment. Letters indicate significant differences determined by Kruskal-Wallis with Dunn post hoc test for treatments under non-drought control, and drought conditions.

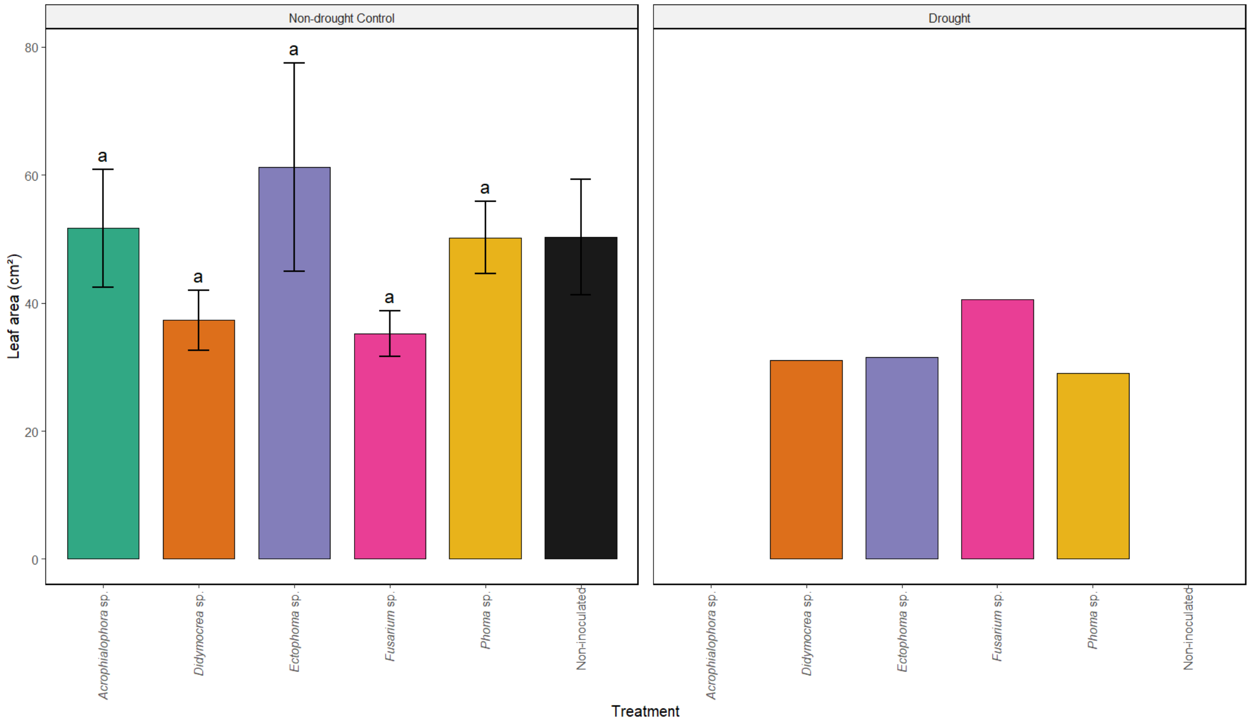

Supplementary Figure 4. Mean new leaf area (cm²) of *Theobroma cacao* ICS95 juveniles exposed to non-drought control (left) and drought conditions followed by rewatering for two weeks (right), measured at the end of the recovery period. Letters indicate significant differences determined by Kruskal-Wallis with Dunn post hoc test for treatments under non-drought control, and drought conditions.

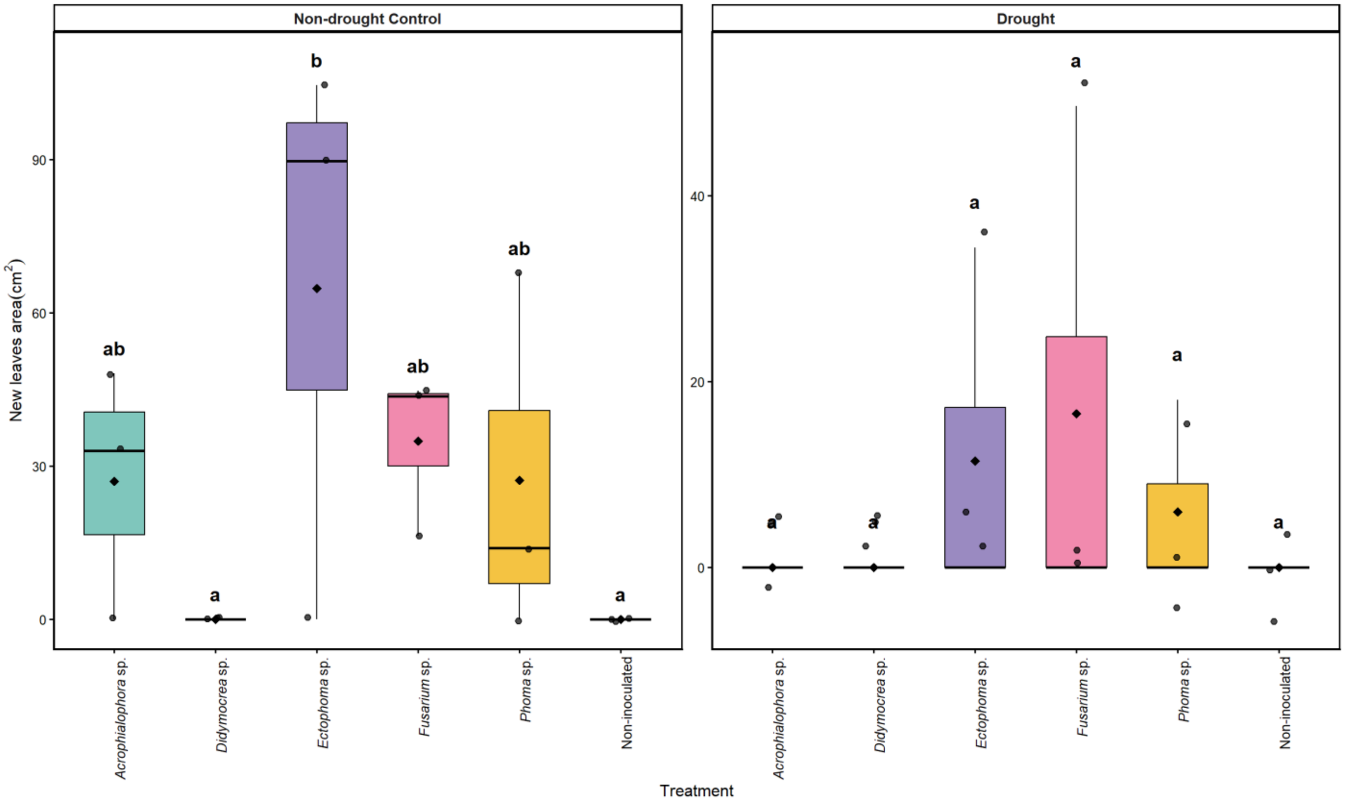

Supplement: Supplemental Material — Tables S1 to S4; Fig. S1 to S4. [file msphere.00865-25-s0001.pdf]
